# Benefits of supportive strategies for carers of people with high-grade glioma: a systematic review: Strategies for addressing the needs of high-grade glioma carers

**DOI:** 10.1007/s00520-022-07419-2

**Published:** 2022-10-25

**Authors:** Diana Jones, Mark B. Pinkham, Matthew P. Wallen, Nicolas H. Hart, Ria Joseph, Esben Strodl, Tamara Ownsworth, Vanessa Beesley, Megan Crichton, Raymond J. Chan

**Affiliations:** 1 Caring Futures Institute, College of Nursing and Health Sciences, Flinders University, Sturt Road, Bedford Park, Adelaide, SA 5042 Australia; 2 Princess Alexandra Hospital, Metro South Hospital and Health Service, Brisbane, QLD Australia; 3 Cancer and Palliative Care Outcomes Centre, School of Nursing, Queensland University of Technology, Brisbane, QLD Australia; 4 School of Science, Psychology and Sport, Federation University Australia, Ballarat, VIC Australia; 5 School of Medical and Health Sciences, Edith Cowan University, Perth, WA Australia; 6 Institute for Health Research, University of Notre Dame Australia, Perth, WA Australia; 7 School of Psychology and Counselling, Queensland University of Technology, Brisbane, QLD Australia; 8 School of Applied Psychology & The Hopkins Centre, Menzies Health Institute Queensland Griffith University, Brisbane, QLD Australia; 9 Supportive Care in Cancer Group, QIMR Berghofer Medical Research Institute, Herston, QLD Australia; 10 Bond University Nutrition and Dietetics Research Group, Bond University, Robina, QLD Australia

**Keywords:** Brain tumour, Glioma, Caregivers, Supportive care

## Abstract

**Purpose:**

To systematically review and examine current evidence for the carer-reported benefits of supportive care strategies for carers of adults with high-grade glioma (HGG).

**Methods:**

Four databases (CINAHL, EMBASE, PubMed, PsycINFO) were searched for articles published between January 2005 and April 2022 that assessed strategies for addressing the supportive care needs of carers of adults with HGG (WHO grade 3–4). Study selection and critical appraisal were conducted independently by three authors (DJ/MC, 2021; DJ/RJ 2022). Data extraction was conducted by one author (DJ) and checked by a second author (RJ). Results were synthesised narratively.

**Results:**

Twenty-one studies involving 1377 caregivers were included, targeting the carer directly (*n* = 10), the patient-carer dyad (*n* = 3), or focused on people with HGG + / − their carers (*n* = 8). A paucity of high-quality evidence exists for effective and comprehensive support directly addressing outcomes for carers of adults with HGG. Strategies that demonstrated some benefits included those that built carer knowledge or provided emotional support, delivered by health professionals or through peer support. Supportive and early palliative care programmes have potential to reduce unmet carer needs while providing ongoing carer support.

**Conclusion:**

Strategies incorporating an educational component, emotional support, and a regular needs assessment with corresponding tailored support are most valued by carers. Future practice development research should adopt a value-based approach and exceed evaluation of efficacy outcomes to incorporate evaluation of the experience of patients, carers, and staff, as well as costs.

## Introduction

Primary brain cancer is a rare (3.5/100,000) [1] yet serious disease. High-grade glioma (HGG), defined as grade 3 and grade 4 glioma by the World Health Organisation (WHO) [2], is most common in adults and is often characterised by the rapid onset and progression of physical and neurological symptoms [3]. Even with standard-of-care treatment comprising surgery followed by radiotherapy and chemotherapy, patients with glioblastoma (WHO grade 4 glioma) have a median overall survival of 12–23 months [4]. Most people with HGG are managed in the outpatient setting, therefore their spouse or other family members usually accept primary responsibility for care [5]. With little time to prepare, carers must learn to navigate the health care system and assume new roles including patient advocate, driver, and medication manager while simultaneously fulfilling pre-existing responsibilities within the family unit [6]. These role changes can lead to significant carer distress, anxiety, and depression [7], with distress continuing throughout the disease trajectory [8] and into bereavement [9].

Carers for people with HGG have unmet needs across a variety of domains, including the need for proactive information [10] and support to manage changing symptoms across the illness continuum [11]. Emotional support is essential as a ‘lifeline’ for carers struggling with grief [12] and the loneliness of caring [13], and acknowledging carer needs recognises and validates their essential role in the caregiving process [14]. To address carers’ unmet needs and improve the provision of supportive care services for people with HGG and their carers, it is essential to identify, synthesise, and evaluate strategies addressing these needs [15].

Several reviews have previously been conducted canvassing supportive care interventions for carers of people with HGG, with no reliable conclusions able to be drawn due to a lack of robust data and adequately-powered studies to evaluate their effectiveness. Sherwood and colleagues [16] undertook a narrative review of neuro-oncology family caregiving. Boele and colleagues [17] conducted a systematic Cochrane review identifying three randomised controlled trials (RCTs) of supportive interventions for carers of adults with HGG [18–20]. Ownsworth and colleagues [21] explored the use of telehealth platforms to deliver supportive care and identified participation by HGG carers in four studies [22–25]. Lastly, a recent systematic review by Heinsch and colleagues [26] examined feasibility and effectiveness of interventions for friends/family of adults with benign and malignant primary brain tumours, with most studies being small-scale or pilot studies (≤ 60 participants). Therefore, conducting another systematic review purely focused on efficacy is likely unfruitful.

Given the rarity of HGG and the complexity of designing and conducting trials with this population [16, 27], it is particularly important to consider what supportive care strategies may be beneficial from the carers’ perspective. To identify supportive strategies valued by carers that address carer-identified needs, and to explore what components of supportive care are most promising to pursue in future research and practice development, this review sought to extend beyond efficacy outcomes and broaden our understanding of strategies helpful for yielding carer-reported benefits. Findings from this review will inform future research for carers of people with HGG, recognising their essential role as partners in the caregiving process.

## Methods

This systematic review was reported in accordance with the Preferred Reporting Items for Systematic Reviews and Meta-analysis (PRISMA) 2020 statement [28] and registered with the International Prospective Register of Systematic Reviews (PROSPERO ID: CRD42021271208).

### Search strategy

**Fig. 1:**
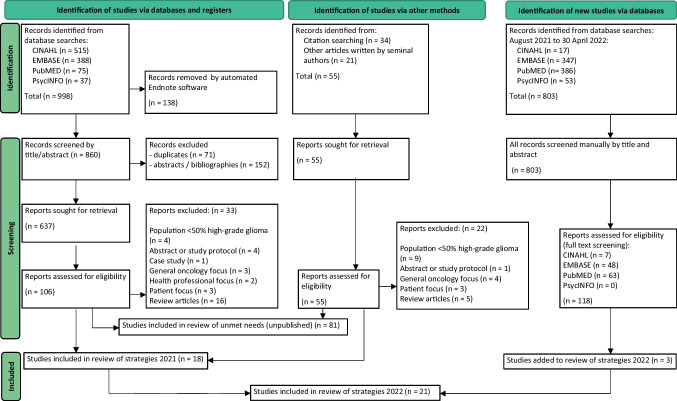
Preferred Reporting Items for Systematic Reviews and Meta-Analyses (PRISMA) diagram for selecting studies that examined the benefit of supportive care strategies for carers of patients with high-grade glioma

Four databases (CINAHL, EMBASE, PubMed, PsycINFO) were searched for studies published from 1 January 2005 to 11 August 2021 using Medical Subject Headings (MeSH) and key words. The systematic search strategy (Fig. 1) used the following: [(high-grade glioma OR glioblastoma OR primary malignant brain tumour OR primary brain tumour OR glioma) AND (carer OR caregiver OR informal carer OR family caregiver)]. Reference lists of included records were screened, and the search strategy repeated 30 April 2022 to update the findings.

### Selection of studies

Two authors (DJ and RJ) independently conducted title, abstract, and full-text screening in duplicate, with disagreements resolved by consensus. Inclusion criteria included [1] adult (> 18 years) carers of adults with HGG, whereby carers were defined as the principal unpaid, informal caregiver; [2] any supportive strategy involving carer participation which reported quantitative and/or qualitative carer data, implemented at any stage of the disease trajectory; [3] described or evaluated any intervention, programme, or service that addressed any carer needs including psychological/emotional needs, information, health service needs, and work/social needs [29]; and [4] included studies of any design published in peer-reviewed journals and written in English. Studies of carers for people with glioma from mixed histologies were excluded if the authors identified < 50% HGG participants. Unpublished articles, theses, pre-prints, trial registries, published study protocols, conference abstracts, and case studies were excluded.

### Review of study quality, data extraction, and synthesis

The quality of included studies was assessed using the Risk Of Bias In Non-randomised Studies-of Interventions (ROBINS-I) assessment tool [30], Revised Cochrane Risk-of-Bias tool for randomised trials (RoB-2) [31], or Mixed Methods Appraisal Tool (MMAT) [32]. Two authors (DJ/MC, 2021; DJ/RJ, 2022) independently critically appraised studies, with disagreements resolved via consensus. The primary outcome was any benefit to carers of people with HGG, with benefits assessed by any outcome measure (e.g. carer knowledge, supportive care activities, quality of life, satisfaction) using any tool. One author (DJ) extracted data relating to study characteristics, participants, strategies utilised, and findings, with data extraction for all studies verified by a second author (RJ). Disagreements regarding data extraction were managed by discussion between authors. Due to the high level of heterogeneity of interventions and types of benefits reported, a meta-analysis was not planned. Data on carer-reported benefits were narratively synthesised under categories of supportive strategies. The narrative synthesis process was consistent with guidance provided by Popay and colleagues [33].

## Results

### Search results and study quality

A total of 21 studies were evaluated (Fig. 1), including eight (38%) non-randomised studies, six (29%) randomised trials, four (19%) mixed-methods, two (10%) qualitative, and one (5%) quantitative descriptive study. All non-randomised studies were assessed to have a serious risk of bias in the selection of participants and reporting of results [34], or by failing to report, or adjust for, confounders [24, 35–40] (Table 1). Four of six randomised studies were assessed to have a high risk of bias, due to the randomisation process [19], deviation from intended intervention [19, 20, 41, 42], or missing outcome data [18–20, 41, 42].

**Table 1:** Quality appraisal of studies assessing supportive care strategies for carers of patients with high-grade glioma

Study & assessment tool	Domains of assessment for risk of bias								Overall quality rating
ROBINS-I (non-randomised studies)	Confounding	Participant selection	Classification of interventions	Deviation from intended interventions	Missing data	Measurement of outcomes	Selection of reported result		
Aoun et al., 2015 (34, 50)	Low	Serious	Low	Moderate	Moderate	Moderate	Serious	Serious risk of bias	
Dionne-Odom et al., 2021 (35)	Serious	Low	Low	Low	Moderate	Moderate	Moderate	Serious risk of bias	
Milbury et al., 2018 (36)	Serious	Low	Low	Low	Low	Moderate	Moderate	Serious risk of bias	
Pace et al., 2014 (37)	Serious	Low	Moderate	Low	Low	Moderate	NI	Serious risk of bias	
Page & Chang, 2016 (38)	Serious	Low	Low	NI	Moderate	Moderate	NI	Serious risk of bias	
Philip et al., 2019 (24)	Serious	Low	Low	Low	Low	Moderate	Moderate	Serious risk of bias	
Pompili et al., 2014 (39)	Serious	Low	Moderate	NI	NI	Moderate	NI	Serious risk of bias	
Wasilewski et al., 2019 (40)	Serious	Critical	Low	Low	Moderate	Moderate	Moderate	Critical	
RoB 2 (randomised trials)	Randomisation	Deviation from intended intervention	Missing outcome data	Measurement of outcome	Selection of reported result			Overall Quality Rating	
Boele et al., 2013 (18)	Low	Low	High	Some concerns	Some concerns			High risk of bias	
Boele et al., 2022 (41)	Low	High	High	Some concerns	Some concerns			High risk of bias	
Locke et al., 2008 (19)	High	High	High	Some concerns	Some concerns			High risk of bias	
Milbury et al., 2019 (47)	Low	Low	Low	Some concerns	Some concerns			Some concerns	
Milbury et al., 2020 (25)	Low	Low	Some concerns	Some concerns	Some concerns			Some concerns	
Reblin et al., 2018 (20, 42)	Low	High	High	Some concerns	Low			High risk of bias	
MMAT (qualitative studies)	1.1. Qual approach appropriate to answer research question?	1.2. Data collection methods adequate to answer research question?	1.3. Findings adequately derived from data?	1.4. Interpretation of results substantiated by data?	1.5. Coherence of data/collection/analysis/interpretation?				
Applebaum et al., 2022 (44)	Yes	Yes	Yes	Yes	Yes			NA	
Halkett et al., 2021 (48)	Yes	Yes	Yes	Yes	Yes			NA	
MMAT (quantitative descriptive studies)	4.1. Sampling strategy relevant?	4.2. Sample representative of target population?	4.3. Measurements appropriate?	4.4. Is the risk of nonresponse bias low?	4.5. Statistical analysis appropriate?				
Catt et al., 2012 (45)	Yes	Yes	Yes	No	Yes			NA	
MMAT (mixed methods studies)	5.1. Rationale for mixed methods design?	5.2. Study components integrated to answer research question?	5.3. Qual/quant components adequately interpreted?	5.4. Differences between quant/qual results addressed?	5.5. Study components adhere to quality criteria?				
Cashman et al., 2007 (43)	Yes	Yes	Yes	Can’t tell	Yes			NA	
Halkett et al., 2018 (23)	Can’t tell	Yes	Yes	Can’t tell	Yes			NA	
Nordentoft et al., 2021 (46)	Yes	Yes	Yes	Can’t tell	Yes			NA	
Piil et al., 2015 (22)	Can’t tell	Yes	Yes	Can’t tell	Yes			NA	

*MMAT*, Mixed Methods Appraisal Tool Version 2018 (32); *RoB 2*, Revised Cochrane Risk-of-Bias tool for randomised trials (31); *ROBINS-I*, Risk Of Bias In Non-Randomised Studies-of Interventions (30)

*Yes*, study meets reporting criteria; *NI*, no information; *NA*, not applicable

Rating key: *Low*, low risk of bias; *Moderate*, moderate risk of bias; *Some concerns*, some concerns as to risk of bias; *High*, high risk of bias; *Serious*, serious risk of bias; Critical, critical risk of bias

### Study characteristics

Twenty-one studies involving 1377 participants from six countries were included: USA (*n* = 10), Australia (*n* = 4), Denmark (*n* = 2), Italy (*n* = 2), and one study each in Canada, Netherlands and UK (Table 2). Three studies used a specialist cancer nurse [23, 24, 35] (*n* = 94 participants), or a neuro-oncology caregiver programme (1 study [38] (*n* = 90 participants). Other strategies included education workshops (2 studies [40, 43] (*n* = 51 participants), meaning-centred psychotherapy (1 study [44] (*n* = 9 participants), and cognitive behavioural therapy or problem-solving strategies (3 studies [18, 19, 41] (*n* = 195 participants). One pilot study assisted carers to identify their social support networks (2 papers [20, 42] (*n* = 40 participants). One study reviewed carer utilisation of a brain tumour website that provided information and on-line support [22] (*n* = 8 participants), and one study compared psychological support with different follow-up care pathways [45] (*n* = 32 participants). Other studies reported on carer participation in a residential rehabilitation programme (1 study [46] (*n* = 16 participants), and evaluated dyadic yoga, exercise, and couple-based meditation (4 studies [25, 36, 47, 48] (*n* = 75 participants). Finally, three studies addressed carer support in the palliative phase of disease [34, 37, 39] (*n* = 767 participants).

**Table 2:** Characteristics, findings and carer needs addressed in the interventions

Study	Participants	Intervention	Outcome Measures	Findings/effectiveness	Carer needs addressed in intervention
Aoun et al., 2015 (34, 50) (Australia) Design: Stepped wedge cluster non-randomised trial	*N* = 29 Gender: Carers: 76% female Patients: 72% male Age: Carers: mean 57 yrs Patients: mean 61 years Caregiver relationship: 86% spouse/partner Time since diagnosis: median 1 yr	Intervention (*n* = 20): Carer needs assessment using CSNAT; shared action plan developed Comparator (*n* = 9): Usual care Setting: Community based palliative care Follow-up: 2–3 weeks post; minimum 2 CSNAT nurse visits Interventionist: Palliative care nurse	Tools: FACQ-PC (strain & distress); SF-12v2 (mental & physical wellbeing); ADLs (workload) Timepoints: Baseline & post intervention	• Intervention groups @ baseline: ↓mental wellbeing **(*p* = 0.010**), ↑strain **(*p* = 0.019**), ↑workload **(*p* = 0.039**) in glioma carers vs other cancers • Post intervention: NS change in glioma carer strain (*p* = 0.833) or mental well-being (*p* = 0.137) pre vs post (no comparison to controls) • Physical wellbeing: Baseline: higher physical wellbeing scores in glioma vs other cancers **(*p* = 0.006)** Post test:↑decline in glioma carers **(*p* = 0.009**) • Limitation: 38% attrition	• Education • Practical assistance • Symptom management • Emotional support • Validation of caregiver role
Applebaum et al., 2022 (44) (USA) Design: Observational	*N* = 9—subset of 60 enrolled in pilot RCT (NCT03454295) Gender: Carers: 100% female Patients: NI Age: Carers: mean 54 yrs Patients: NI Caregiver relationship: 78% spouse/partner Time since diagnosis: mean 8 caring months	Intervention: 7 sessions of Meaning-Centered Psychotherapy for Cancer Caregivers (MCP-C) either in person (*n* = 5) or via telepsychiatry (*n* = 4) Comparator: Enhanced Usual Care (distress screening + targeted referrals) Setting: Memorial Sloan Kettering Cancer Centre Interventionist: Clinical psychology graduate students & fellows	Tool: Semi structured interview Timepoint: Post intervention	• Carer Feedback on MCP-C: • Allowed carers to reflect on caregiving experience • Reframe caregiving as a part of (not entire) self-identity • Recognition of ‘attitude’ in how individual reacts to stressors • Importance of self-care • MCP-C most beneficial early in patient’s illness (mean of 8 months of caring at time of intervention)	• Validation of role • Acknowledgment of own needs • Meaning making in caregiving
Boele et al., 2013 (18) (Netherlands) Design: Randomised control trial	*N* = 56 Gender: Carers: 74% female Patients: 71% male Age: Carers: mean age 51 yrs Patients: mean age 53 yrs Care relationship: not specified (‘spouse or significant other’) Time since diagnosis: 66% < 1 yr	Intervention (*n* = 31): 6 × 1 h of Cognitive Behavioural Therapy sessions Comparator (*n* = 25): Usual care Setting: Tertiary referral centre for neuro-oncology patients Interventionist: Psychologist	Tools: HRQOL (SF-36); Caregiver mastery scale Timepoints: Baseline & every 2 months = 5 in total	• Mastery increased over time **(*p* = 0.021)** (after adjusting for confounders) vs usual care group • Mental functioning stable for int vs ↓ usual care (not sig after adjusting for confounders (*p* = 0.113)) • Limitation: 52% attrition intervention vs 32% control	• Symptom management • ‘Patient contact’ issues • ‘Contact with family/friends’ • Time for self
Boele et al., (2022) (41) (USA) Design: Randomised control trial	*N* = 120 Gender: Carers: 64/76% female Patients: 63/70% male Age: Carers: mean 53/52 yrs Patients: mean 55/53 yrs Care relationship: 76% spouse Time since diagnosis: within 4 months of initial diagnosis or recurrence	Intervention: (*n* = 80) 8 week needs-based support programme ‘SmartCare’ (problem-solving) + / − self-guided CBT ‘Beating the Blues’ Comparator (40): Enhanced care as usual (ECAU) Setting: Online programmes Interventionist: Nurse-led	Tools: CED-D; CNS; POMS-Anxiety; CMS (Mastery); CRA Timepoints: Baseline & 2, 4, 6 & 10 months	• Accrual lower than expected: > 2 intervention arms combined, 58% initiated SmartCare • Reduction in caregiving-specific distress scores vs ECAU for intention to treat **(*p* = 0.012)** & per protocol analysis **(*p* = 0.021)** • No intervention effect on depression, anxiety, or burden; trend in intervention group toward mastery • Carer feedback: preferred autonomy when choosing when to engage in intervention • Limitation: Attrition rates intervention (36%) vs control (13%)	• Problem-solving strategies for carer-identified needs
Cashman et al., 2007 (43) (Canada) Design: Observational	*N* = 24 Gender: Carers: 80% female Patients: NI Age: Carers: 33% 46–55 yrs Patients: NI Care relationship: 50% spouse Time since diagnosis: NI	Intervention (*n* = 24): 2 × half-day education sessions, Question & Answer periods, group discussion Comparator: Nil Setting: Hospital Interventionist: Neuro-oncologist, Advanced practice nurse, Palliative physician, Occupational therapist, Social worker, Neuropsychologist	Tool: Multiple choice test questions assessing knowledge related to programme content Timepoints: Baseline & post intervention; post test repeated at 4–6 weeks	• Improvement in knowledge scores post intervention **(*p* = 0.05)** • 4–6 weeks post: mean scores ↓ but statistically higher vs baseline = information retention • 25% wanted information earlier in caregiving experience • 71% reported benefit of sharing experiences with other carers	• Validation of caregiver role • Building caregiver knowledge & skills • Symptom management • Managing behavioural & cognitive changes • Psychosocial support (including peers) • Dealing with uncertainty • Time for self
Catt et al., 2012 (45) (UK) Design: Observational	*N* = 32 Gender: Carers: 50/60% female Patients: 52/53% female Age: Carers: mean 54/46 yrs Patients: mean 55/46 yrs Care relationship: 85/92% spouse/partner Time since diagnosis: 6 months	Intervention 1 (*n* = 20): Oncologist-led follow up Comparator (*n* = 12): Multidisciplinary group follow up Setting: Hospital outpatient clinic (interviews conducted as home visit) Interventionist: Researcher	Tools: GHQ-12; questionnaire Timepoints: Baseline, 3 months, 6 months	• ↑carer anxiety vs patients for clinic visits (6/10 vs 4/10) & scans (5/10 vs 3/10) • ↑psychological morbidity assoc with female, not a spouse, childcare responsibilities, greater lifestyle adjustments • 56% carers had probable psychological morbidity @ baseline, wellbeing not influenced by follow up method • Limitation: 40% attrition (77% lost to follow up had probable psychiatric morbidity)	• Inadequate psychological support with either method of follow up • Carers identified need for proactive nurse telephone follow-up & information about course of illness
Dionne-Odom et al., 2021 (35) (USA) Design: Observational	*N* = 53 Gender: Carers: 62% female Patients: 59% male Age: Carers: mean age 54 yrs Patients: mean age 52 yrs Care relationship: 79% spouse/partner Time since diagnosis: ‘recently diagnosed’	Intervention (*n* = 53):‘FamilyStrong’: clinic-based telehealth support service Comparator: Nil Setting: Neuro-oncology outpatient dept or telephone screening Interventionist: Palliative care nurse	Tools: DT; PHQ-2; GAD-2; caregiver & patient resource use Timepoints: Monthly distress screening; 3 monthly comprehensive assessment	• Distress screenings (*n* = 235): 46% moderate distress*/*13% high distress • Difference between ‘bothersome problems’ & those that carers wanted assistance with • 211 interventions to provide emotional support*/*information*/*facilitate communication*/*problem-solving*/*internal & external referrals	• Information*/*symptom management • Coordinating services*/*practical help • Planning for future • Emotional support (sadness) • Knowing when to seek help • Communicating with treating team • Finances/insurance
Halkett et al., 2018 (23) (Australia) Design: Observational	*N* = 10 Gender: Carers: 50% female Patients: 60% female Age: Carers: 56 yrs Patients: 56 yrs Care relationship: 70% spouse/partner Time since diagnosis: < 6 months	Intervention (*n* = 10): ‘CARE-IS’: Telephone assessment of needs; Tailored resource folder; Nurse-led home visit; Monthly phone support × 6 months Comparator: Nil Setting: Home*/*telephone screening Interventionist: Neuro-oncology research nurse	Tools: PCS; DT; HADS; CQOLC; Carer Competence Scale; CSI (modified); SCNS-P&C; Brain Tumour Specific SCNS; Healthcare utilisation Timepoints: Baseline, 6 & 12 weeks	• Feasibility: 83% accrual • 79 episodes of referrals*/*provision of additional information • Resource manual expanded with added info based on participant feedback • Mean distress @ baseline 4.9/10, NR post intervention • Limitation: 30% attrition @ 3 months	• Caring for yourself • Financial/legal concerns • Dealing with treatment • Understanding physical symptoms*/*mental & behavioural changes • Lifestyle choices • Fertility & sexuality • End of life questions
Halkett et al., 2021 (48) (Australia) Design: Observational	*N* = 15 carers (20 patients) Gender: Carers: NI Patients: NI Age: Carers: NI Patients: mean age 53 yrs Care relationship: 70% dyads Time since diagnosis: 70% < 3 months	Intervention: (*n* = 15) Group session (up to 5 participants) 60-min supervised, individually tailored exercise, 3 sessions/week for 7 weeks Comparator: Nil Setting: Undertaken while receiving radiation treatment Interventionist: Exercise physiologist	Tools: Semi-structured telephone interview Timepoints: Post intervention	• Carer benefits: • Mutual motivation by doing the session together • Opportunity to ‘take time out’ while patient doing the session, reassurance that patient was in a safe environment • Focus on something other than the disease • Challenges: juggling other commitments*/*appts to make time for sessions	• Self-care (either through participation or opportunity to ‘take time out’
Locke et al., 2008 (19) (USA) Design: Randomised controlled pilot	*N* = 19 Gender: Carers: NI Patients: 58/57% male Age: Carers: NI Patients: mean age 50/57 Care relationship: NI Time since diagnosis: 74% within 2 months	Intervention (*n* = 12): 6 sessions cognitive rehab & 6 × problem-solving (concurrent), over 2 weeks Comparator (*n* = 7): Usual care Setting: Undertaken while receiving radiation treatment*/*telephone post intervention Interventionist: Neuro-psychologist or Master’s level Behavioural therapist	Tools: Post-Study Feedback Questionnaire; LASA scale; CQOLC; POMS Timepoints: Baseline, 2 weeks (post intervention) & 3 months	• Carer QOL: No intervention effect vs control (↑ CQOLC in controls, p values NR) • Mood: No intervention effect vs control (p values NR) • 88% carers reported intervention ‘very or somewhat helpful’ • Limitation: 33% attrition	• NI
Milbury et al., 2018 (36) (USA) Design: Observational	*N* = 5 Gender: Carers: 60% female Patients: 80% female Age: Carers: mean age 58 yrs Patients: mean age 52 yrs Care relationship: 60% spouse Time since diagnosis: < 7 months	Intervention (*n* = 5): 12 × 60-min dyadic yoga, 2–3 sessions/week Comparator: Nil Setting: Undertaken while receiving radiation treatment Interventionist: Certified yoga instructor, experienced in working with cancer patients	Tools: CES-D; BFI; PSQI; SF-36; programme evaluation Timepoints: Baseline (1^st^ week of radiotherapy) & last week of radiotherapy, weekly programme eval	• Feasibility: 100% retention and adherence • 67% ‘very useful’, 33% ‘useful’ • Improvement (medium effect) in caregiver mental QOL (Cohen’s *d* = 0.64) • Marginally significant increase in depression scores (Cohen’s *d* = 1.04)	• Mental health*/*quality of life
Milbury et al., 2019 (47) (USA) Design: Randomised controlled pilot	*N* = 20 Gender: Carers: 70/60% female Patients: 50% female Age: Carers: mean age 50 yrs Patients: mean age 46 yrs Care relationship: 60–50% spouse Time since diagnosis: < 7 weeks since diagnosis	Intervention (*n* = 10): 12 × 45-min dyadic yoga, 2–3 sessions/week Comparator (*n* = 10): Wait-list control group Setting: Undertaken while receiving radiation treatment Interventionist: Certified yoga instructors experienced in working with oncology patients	Tools: CES-D; BFI; SF-36 Timepoints: Baseline (1^st^ week of radiotherapy) & last week of radiotherapy	• Feasibility: 70% accrual • Adherence: 88% (mean of 10.6 sessions completed) • Retention: 95% completed all surveys • Baseline: 35% ‘caseness’ for carer depression, higher caseness scores intervention vs control (*p* ≤ 0.05) • Post intervention: Improvement in depressive symptoms (Cohen’s *d* = 1.12 (large effect), ***p* = 0.03**), fatigue (*d* = 0.89, *p* = 0.07) & mental wellbeing (*d* = 0.49) vs wait-list controls • 100% rated programme ‘useful’ or ‘very useful’	• Mental health*/*quality of life
Milbury et al., 2020 (25) (USA) Design: Randomised controlled pilot	*N* = 35 Gender: Carers:57% female Patients: 54% male Age: Carers: mean age 53 yrs Patients: mean age 57 yrs Care relationship: 100% spouse/partner Time since diagnosis: < 12 months	Intervention (*n* = 18): 4 × 60-min weekly couple-based meditation (mindfulness, compassion for self & partner, gratitude, values-based living Comparator (*n* = 17): Usual care Setting: delivered by FaceTime (audio-visual interaction) Interventionist: Psychology counsellor intern (master-level)	Tools: DT; CES-D; Mindful Attention Awareness Scale; Self-Compassion Scale; Personal Assessment of Intimacy in Relationships Inventory Timepoints: Baseline, 6 & 12 weeks	• Feasibility: 62% accrual • Adherence: 67% all sessions, 78% minimum 3 sessions • 100% rated sessions ‘beneficial’ or ‘very beneficial’ & overall programme ‘useful’ or ‘very useful’ • Baseline: 27% carers had CES-D caseness • No intervention effect for carers vs control on any of the measures: depression, mindfulness, compassion, intimacy (p values NR) • Limitation: 37% attrition	• Mental wellbeing support
Nordentoft et al., 2021 (46) (Denmark) Design: Observational	*N* = 16 Gender: Carers: 68% female Patients: 53% male Age: Carers: median age 60 yrs Patients: median age 60 yrs Care relationship: 81% spouse Time since diagnosis: < 12 months (*n* = 6), 1–2 yrs (*n* = 5), > 2 yrs (*n* = 6)	Intervention (*n* = 16): 4-day residential rehabilitation programme & 2-day follow-up programme @ 3 months; Carer only group discussion & Mental reactions/mindset sessions Comparator: Nil Setting: Residential programme @ Rehab & Palliative Care Centre Interventionist: Neuro-psychologist, physician, nurse, social worker, physiotherapist, occupational therapist, massage therapist Carer-specific content led by neurosurgical nurse and Centre for Brain Injury representative	Tools: Questionnaire; dyad interview Timepoints: Questionnaire after each session; evaluation @ end of each programme; dyad interviews @ start of follow-up programme & last day	• Overall satisfaction for initial programme 4.8/5, 4.3/5 for follow-up • 91% completed initial programme • Opportunity for peer support beneficial • Importance of individualised information • Group therapy beneficial & created a positive dynamic • Action plan difficult to implement → stress → sense of defeat • Limitation: 44% attrition	• ‘Safe space’ for caregivers to meet peers without patient being present • Unpreparedness for caregiving role • Guidance for carers not living with patient • Information earlier in illness trajectory
Pace et al., 2014 (37) (Italy) Design: Observational	*N* = 616 Gender: Carers: NI Patients: NI Age: Carers: NI Patients: NI Care relationship: NI Time since diagnosis: NI, ‘terminal phase of disease’	Intervention (*n* = 616): Comprehensive home-based palliative care; interventions based on disease progression: low intensity (weekly visit/phone call) to > high (min. 3 visits/week) Comparator: Nil Setting: Home Interventionist: Neurologist, nurse, psychologist, rehabilitation therapist, social worker	Tools: ‘Customer satisfaction’ survey to assess carer perception of quality of care received Timepoints: Not specified (‘periodically’)	• Programme provided psychological support for carers during entire course of disease, including bereavement support • Nurse as case manager liaised with GP*/*District Health Service for continuity of care • Carer satisfaction: Home-care assistance 98%; Communication 93%; Nursing 95%; Home rehab 92%; Social work help 88%	• Carer training to manage physical disabilities • Proactive info to anticipate symptoms & problems • Emotional support, including bereavement support • Advance care planning to identify end-of-life preferences
Page & Chang, 2016 (38) (USA) Design: Observational	*N* = 90 Gender: Carers: NI Patients: NI Age: Carers: NI Patients: NI Care relationship: NI Time since diagnosis: Newly diagnosed	Intervention (*n* = 90): Clinic-based neuro-oncology caregiver programme: 4 proactive phone calls; monthly in-person support group; telephone support group; matched peer-to-peer carer support Comparator: Nil Setting: Neuro-oncology clinic Interventionist: Neuro-oncology nurse, social worker, administrative coordinator/analyst	Tools: Screening & needs assessment tool; survey; assessment flowsheet added to patient’s electronic medical record Timepoints: Baseline, 2, 4, 8 weeks & 4 months	• Interventions delivered: education, carer access to resources, practical & emotional support, referrals, peer support • Highest needs for emotional support & advocacy assistance (> 30%) • Survey results of initial 197 carers (25% response): • Needs & timing of desired assistance vary, need to assess carer readiness for information & support • Repeated outreach calls improve knowledge & carer satisfaction	• Emotional support • Disease-specific info • Advocacy support for legal, insurance, employment, financial issues • Navigating family dynamics e.g. parenting & marital concerns • Access to caregiver resources • Opportunities for peer support
Philip et al., 2019 (24) (Australia) Design: Observational	*N* = 31 Gender: Carers: 58% female Patients: 53% male Age: Carers: mean age 56 yrs Patients: mean age 61 yrs Care relationship: 81% spouse/partner Time since diagnosis: Newly diagnosed	Intervention (*n* = 31): Structured supportive care ‘I-Cope’ (Information Coordination Preparation Emotional) Elements: staged information, regular screening for needs, communication and coordination, family carer engagement Comparator: Nil Setting: Neuro-oncology tertiary hospital (face-to-face initial contact then telephone) Interventionist: Cancer care coordinator	Tools: DT; CSNAT; PINQ; CQOLC; PCS Timepoints: Baseline, 2 & 12 weeks	• Feasibility: 86% accrual • Retention: 94% carer completion • ‘High’ satisfaction with communication, ‘very high’ confidence & trust in care team • 87% overall care ‘excellent’ (not specific to I-CoPE) • Reduction in carer information needs **(*p* = 0.002)** & unmet supportive care needs **(*p* = 0.019)**, ↑ preparedness to care (***p* = 0.043**) • Duration total interactions/carer = 69 min (range 45–130 min) • Cost analysis of intervention AUD$137 per dyad	• Information • Emotional support • Value of supportive relationship with care coordinator • Regular screening to respond to emergent needs
Piil et al., 2015 (22) (Denmark) Design: Observational	*N* = 8 Gender: Carers: NI Patients: 64% female Age: Carers: NI Patients: median age 64 yrs Care relationship: NI Time since diagnosis: Newly diagnosed	Intervention (*n* = 8): Evaluation of newly developed Brain tumour website (BTW) Features: HGG info/medical terminology, website links, ‘ask-the-specialist’ feature, online support group, phone access to moderator Comparator: Nil Setting: Web-based service Interventionist: Moderator & specialists from neurosurgery, neurology, oncology, rehabilitation & palliative care social worker	Tools: Nationwide usage survey; semi-structured telephone interview Timepoints: Nationwide survey @ 6 months, carer interview @ 3 months	• Nationwide survey: BTW accessed by 637 devices, 19 carers logged on, 4 carers contributed personal stories • Telephone support line rarely used • 37% carers in study (*n* = 3) had accessed site to ask specialist questions or share experiences • Barriers to use: age, technological challenges, inadequate detail in answers provided	• Tailored information when participants indicate they are ready to receive it
Pompili et al., 2014 (39) (Italy) Design: Observational	*N* = 122 Gender: Carers: NI Patients: NI Age: Carers: NI Patients: NI Care relationship: NI Time since diagnosis: ‘Care of patient after discharge from initial surgery to end-of-life’	Intervention (*n* = 122): Home-based palliative care; Intensity of intervention based on disease progression: low intensity (weekly visit/phone call) to > high (minimum 3 visits/week) Comparator: Nil Setting: Home Interventionist: Neurologist, nurse (case manager), psychologist, rehabilitation therapist, social worker	Tools: ‘Customer satisfaction’ survey (self-administered) to assess carer perception of quality of care received Timepoints: Monthly survey	• Survey results for carer satisfaction: 97% home assistance, 95% nursing care, 90% communication, 92% rehab at home, 85% social work help • Higher satisfaction rate when carer actively involved • Care plan involved GP & District Health Services to provide 24-h support	• Education and training for caregivers in physical care, e.g. positioning, mobilising, pressure area prevention • Psychological support for the whole family
Reblin et al., 2018 (20, 42) (USA) Design: Randomised control trial	*N* = 40 Gender: Carers:75% female Patients: 53% male Age: Carers: mean age 57 yrs Patients: mean age 52 yrs Care relationship: 64% spouse Time since diagnosis: Mean of 32 months	Intervention (*n* = 30): Electronic Support Network Assessment Program (eSNAP) to identify sources of carer support: hands-on, informational, communication, financial, emotional, self-care Comparator (*n* = 10): Usual care Setting: Electronic web-based tool Interventionist: Carer participation	Tools: Zarit Caregiver Burden Scale; HADS Timepoints: Baseline, 3 & 6 weeks, Initial questionnaire during clinic visit, then online at home	• 80% accrual, 80% completion • No intervention effect vs control for carer anxiety (*p* = 0.380), burden (*p* = 0.617) or helpfulness of social support (*p* = 0.945) • Depression levels remained high but stable in intervention group, increased in controls (*p* = 0.072) ?protective effect • 80% did not refer back to eSNAP results (given as PDF) at follow-up reviews; no means to revisit results using the app	• Proactive psychosocial support • Identify & evaluate carer support networks • Carers with poor support networks to be ‘flagged’ for social worker referral
Wasilewski et al., 2019 (40) (USA) Design: Observational	*N* = 27 Gender: Carers: 70% female Patients: 65% male Age: Carers: median age 59 yrs Patients: median age 60 yrs Care relationship: 74% spouse Time since diagnosis: NI	Intervention (*n* = 27): Education session: seizure recognition, safety, home management Comparator: Nil Setting: Hospital neurology department Interventionist: Neuro-oncologist	Tools: Pre/post knowledge test; DT (seizure-related distress) Timepoints: Baseline & post intervention, 2 & 6 months	• 74% completion • Baseline: higher carer distress (5/10) vs patients (2.5/10) despite better seizure knowledge • NS change in median distress post intervention (4/10), distress range 0–9 pre vs 0–8 post: > suggests some carers remained highly distressed • Knowledge increased & sustained post intervention (baseline 7/9, 2 months 8/9, 6 months 8.5/9), *p* values NR • No acute presentation for seizures in 6 months post study	• Education to improve carer confidence to manage symptoms • Education early in disease trajectory •Need to acknowledge carer distress in relation to seizures (concern for disease progression

Abbreviations: *ADLs*, activities of daily living; *BFI*, Brief Fatigue Inventory; *CDC*, Centers for Disease Control and Prevention; *CES-D*, Centre for Epidemiological Studies-Depression; *CMS*, Caregiver Mastery Scale; *CNS*, Caregiver Needs Screen; *CQOLC*, Caregiver Quality Of Life Index-Cancer; *CRA*, Caregiver Reaction Assessment; *CSI*, Caregiver Strain Index; *CSNAT*, Carer Support Needs Assessment Tool; *DT*, Distress Thermometer; *FACQ-PC*, Family Appraisal of Caregiving Questionnaire; *GAD-2*, General Anxiety Disorder-2; *GHQ-12*, General Health Questionnaire; *HADS*, Hospital Anxiety and Depression Scale; *HRQOL*, health-related quality of life; *LASA*, Linear Analogue Self-Assessment scale; *NI*, no information; *NR*, not reported; *NS*, not significant; *PCS*, Preparedness for Caregiving Scale; *PHQ-2*, Patient Health Questionnaire-2; *PINQ*, Patient Information Needs Questionnaire; *POMS*, Profile Of Moods Scale; *PRISM*, Program for Readability in Science & Medicine; *PSQI*, Pittsburgh Sleep Quality Index; *SCNS-P&C*, Supportive Care Needs Survey-Partners and Caregivers; *SF-36*, Medical Outcomes Study 36-item Short Form Survey; *SF-12v2*, Short Form survey, version 2; *yr*, year

Benefits to carers were measured by the number and type of supportive activities undertaken during the study [22, 23, 35, 38], carer feedback [34, 43, 44, 46, 48], or by a change in physical or mental wellbeing [20, 25, 34, 36, 41, 45, 47], quality of life [19], caregiver strain [34], or burden [41], mastery [18, 41], knowledge [40, 43], preparedness to care [24], or satisfaction [37, 39, 46]. Significance was assumed if *p* < 0.05 (Table 2).

### Participant characteristics

Most carers were female (50–100% of participants), with a mean age range of 50–60 years, and identified as the spouse or partner (range 50–92%). People with HGG were predominantly male in twenty studies (50–72% of participants), with a mean age range of 46–64 years, and included those with a recent diagnosis through to a terminal phase of disease. Several studies did not report carer demographics [19, 22], demographics of people with HGG [43, 44], or demographics for either group [37–39].

### Supportive strategies: information/education/problem-solving

Carers reported benefit across several domains by attending education workshops. Knowledge scores improved after attending a neuro-oncology workshop focused on carer-identified topics including disease and treatment options, symptom management, and strategies to manage cognitive changes (*n* = 24) [43]. Carers positively evaluated the programme meeting their needs regarding content, location, and timing, although 25% would have liked the information earlier in the care trajectory [43]. Sharing experiences with other carers was highlighted as beneficial by 71% of participants, while others valued talking informally with healthcare staff and being acknowledged as the primary carer [43]. Similarly, an education session on tumour-related epilepsy demonstrated an increase in carer knowledge, although the need for information earlier was again reported (*n* = 27) [40]. Carers highlighted that the intervention reduced their worry, and increased their confidence and preparedness to manage seizures [40].

Conversely, 63% of carers did not access a brain tumour website providing tailored information and on-line support (*n* = 8) [22]. One ‘non-user’ acknowledged the value of the service, and two carers utilised the ‘ask the specialist’ service; however, other feedback suggested a lack of confidence in internet information meeting their individual needs, and a perception that web-based interventions were better suited to younger people.

A recent three-arm RCT of a problem-solving intervention (+ / − cognitive behavioural therapy (CBT) for depression) demonstrated a significant reduction in caregiving-specific distress compared to ‘enhanced care as usual’ (*n* = 120) [41]. ‘Smart Care’ involved an online needs-based assessment and goal-setting strategy with nurse support. The intervention arms were combined due to poor CBT accrual; however, only 58% of carers initiated a needs assessment and plan, with higher attrition in the intervention group (36%) compared to control (13%). While carers noted the intervention to be helpful, feedback also highlighted the importance of carer autonomy in choosing when to engage in a supportive strategy [41]. In comparison, a dyadic cognitive rehabilitation and problem-solving RCT (*n* = 19 dyads) [19] was also evaluated by carers as ‘helpful’; however, it demonstrated no significant improvement in carer quality of life or mood status compared to usual care [19].

### Supportive strategies: peer/psychological/social support

The value of peer support was highlighted by carers (*n* = 16) participating as dyads in a residential rehabilitation programme [46]. Carers valued having a ‘safe space’ to discuss issues without the patient being present, and support from other carers in sharing personal experiences of challenging situations [46]. Similarly, the opportunity to reflect on, and find meaning in the caregiving experience was reported as beneficial by carers participating in a psychotherapy study (*n* = 9) [44]. In contrast, the evaluation of follow-up care pathways for people with HGG (*n* = 40) and carers (*n* = 32) concluded that neither pathway had an impact on carer psychological wellbeing [45]. While carers reported satisfaction with both pathways, the need for emotional support was identified as the primary recommendation for service improvement.

A significant increase in mastery was reported from a RCT involving CBT and psychoeducation (*n* = 56) [18]. Carers highlighted needing support to manage patient-related concerns, deal with family/friends, and find time for themselves. However, there was higher attrition in the intervention group (52%) compared to controls (32%), suggesting that study participation was likely too burdensome for participants.

The impact of complementary therapies on mental wellbeing was explored in several studies. Milbury and colleagues [36] conducted a pilot of dyadic yoga (*n* = 5 dyads), followed by a pilot RCT (*n* = 20 dyads) [47]. Participants reported the intervention ‘useful’ and ‘beneficial’, with significantly less carer depressive symptoms compared to waitlist control [47]. Similarly, carers participating as dyads in a tailored exercise programme (*n* = 15) noted the value of mutual motivation, and focusing on something ‘other than the cancer’ [48]. The opportunity to ‘take time out’ while the person with HGG was in a safe and supportive programme was also valued, although challenges in coordinating appointments were noted [48]. Carer feedback from a pilot RCT of couple-based meditation (*n* = 35 couples) rated the components ‘beneficial’, the programme ‘useful’, and would recommend it to other couples [25]. Participants valued the dyadic format; however, there were no significant group differences in carer depressive symptoms, mindfulness, compassion, or intimacy compared to usual care.

‘Eco-mapping’ social support networks was evaluated in a randomised pilot study for 40 carers using an electronic Social Network Assessment Program (eSNAP) [20, 42]. Carers reported the phone application was easy to understand and helped them consider their support networks. However, only 20% referred back to the ‘eco-map’ at subsequent reviews, and there was no significant intervention effect on carer anxiety, burden, or helpfulness of support compared to usual care.

### Supportive strategies: comprehensive support: specialist nurse-led programmes

The ‘Care-IS’ pilot programme included a needs assessment, nurse-led home visit, tailored resource manual, and monthly phone support (*n* = 10) (23). Carers reported the manual’s information was helpful and understandable; however, feedback also identified the need for additional information which was added to the resource manual. Although carers were recruited within two months of diagnosis, 40% would have preferred the information earlier in the disease trajectory. There were 79 documented episodes of information provision/referrals, with carers needing support to manage symptoms, mental and behavioural changes, financial concerns, and anxiety and distress.

Philip and colleagues [24] reported on the non-randomised ‘I-CoPE’ study where cancer care coordinators provided staged information, regular needs screening, and emotional support for people with HGG (*n* = 32) and their carers (*n* = 31). The intervention targeted three transition points: after diagnosis, following hospital discharge, and after completion of radiotherapy. Results demonstrated a significant reduction in unmet information and supportive care needs, and increased preparedness to care [24]. Carer distress declined over time, though remained at clinically relevant levels throughout the intervention [49]. Most carers (87%) rated overall care to be ‘excellent’, including ‘high’ satisfaction with communication and ‘very high’ confidence and trust in the care team [24]. Carer-reported needs changed from an early focus on practical concerns regarding treatment and care responsibilities, to later needs focused on managing the emotional impact on children and family coping.

‘FamilyStrong’ offered a programme of nurse-led telehealth support for 53 carers of people with newly-diagnosed HGG [35]. Providing emotional support and education were the most frequent activities from the 211 documented encounters, with 59% of carers reporting moderate or high distress. Carers distinguished between problems that ‘bothered them the most’, as distinct from issues they wanted ‘assistance to manage’ which included managing their relative’s health condition/symptoms, coordinating care/services, sadness, and planning for the future.

### Supportive strategies: comprehensive support: clinic-based caregiver programme

The Neuro-Oncology Gordon Murray Caregiver Program at the University of California, San Francisco, provided regular phone reviews, peer support, support groups, and an annual neuro-caregiver workshop [38]. Carer-reported benefits included increased knowledge and emotional support from sharing concerns and discussing coping strategies. Feedback also highlighted the value of repeated screening and outreach calls to improve satisfaction with care, and identify carer readiness to accept help. Supportive care needs changed over time and included the need for emotional support, advocacy assistance, and support to address family coping.

### Supportive strategies: comprehensive support: palliative phase

Aoun and colleagues [34] analysed a sub-set of HGG carers (*n* = 29) from a community-based palliative care trial [50]. Results from the wider trial demonstrated a significant reduction in caregiver strain compared to controls [50], with no significant effect for HGG carers. Carer needs changed over time, with ‘knowing what to expect in the future’ remaining the highest reported need, and ‘managing your relative’s symptoms’ increasing from baseline. Qualitative feedback highlighted that regular needs screening provided reassurance, helped empower carers to find solutions and encouraged them to reflect on the emotional impact of caregiving. While this intervention targeted the end-of-life home-based phase of care, carers noted it would have been beneficial earlier in the disease trajectory [34].

A programme of early comprehensive palliative home care [37] was also positively valued by carers (*n* = 616), with high satisfaction rates across all support domains including home-care assistance (98%), nursing (95%), communication (93%), rehabilitation at home (92%) and social work help (88%). Hospital re-admission rates were lower in the last two months of life compared to a similar cohort of glioblastoma patients (16.7% vs 38%), which helped maintain carer support into bereavement. A later cohort of participants in this programme (*n* = 122) also reported high satisfaction rates across all domains [39] and identified that education, including learning physical care strategies, allowed carers to be more involved in the care of their loved one and increased carer satisfaction.

## Discussion

This systematic review synthesises current evidence of carer-reported benefits from strategies to address supportive needs of carers for people with HGG. As expected, we found limited evidence of the efficacy of supportive strategies. Instead of focusing on efficacy alone, this review took a slightly different approach to identify and synthesise carer-reported benefits. This approach is more likely to allow clinicians and researchers to incorporate strategies valued by carers as the evidence base in this area continues to evolve. Overall, strategies that provided peer support [43, 46] and emotional support from health professionals [34, 38, 43] appeared to be highly valued by carers. Supportive and early palliative care strategies appeared to reduce carers’ unmet needs, increase preparedness to care, and provide support for the whole family [24, 37, 39]. Educational strategies such as workshops [19, 42] and individualised resources [23] also demonstrated some carer-reported benefits and are potential strategies for building carer knowledge and skills to manage symptoms.

It is noteworthy that several studies failed to report meaningful qualitative data to build our understanding of carer benefit [35]. For example, carers evaluated programmes to be ‘helpful’ and ‘useful’ [19, 20, 25] without any further qualitative exploration of benefit, or demonstrating intervention efficacy. It is uncertain whether this discrepancy could be due to positive response bias, a placebo effect of participating in any supportive strategy [20], or whether carers derived an added benefit from the intervention beyond the pre-determined outcome measures (e.g. peer support from other participants) [43]. Prioritising qualitative data collection using a mixed-methods approach, including carer perception of benefit, alongside the assessment of quantitative outcomes is congruent with the recommendations of Heinsch and colleagues [26] for future research to reflect the caregiver experience of participating in supportive care strategies.

Supportive strategies valued by carers were those that incorporated some element of emotional support [34, 38]. Peer support was perceived as valuable in providing a ‘safe space’ for carers to discuss sensitive issues and share experiences [43, 46]. Emotional support from health professionals helped to validate the carer’s role and reduce isolation [34, 43]. ‘Flagging’ issues with the nurse, even in the absence of a ready solution, was also reported to be beneficial [34]. While most strategies built rapport with face-to-face interactions [24], telehealth services offered the advantage of ongoing support at a time convenient for the clinician and carer [35].

Strategies that provided carer education were valued by carers to improve knowledge, confidence, and preparedness to care [40, 43]; however, carer feedback identified the need for knowledge earlier in the illness continuum [23, 34, 40, 43, 46]. Previous research has highlighted that timing of information provision is crucial, with too much information potentially overwhelming the carer, and too little leaving them unprepared [6, 51, 52]. This finding was echoed in the ‘Care-IS’ study, with some carers wanting comprehensive information immediately after diagnosis, whereas others recognised they would be unable to process it [23]. While timing and delivery of information can vary depending on individual carer preferences, it remains that timely education and upskilling of carers is a proactive measure that can help build carer competence and confidence [10, 13, 38, 40].

In the included studies, the principal areas of carer-reported need focused on knowledge and symptom management [23, 24, 34, 35, 38–40, 43] alongside emotional support [23, 24, 34, 35, 38, 45, 46], with added concerns regarding the impact of a HGG diagnosis on children and family coping [18, 24, 37, 38]. Other carer-identified needs included understanding mental and behavioural changes [23, 43], coordinating services [23, 35, 38], and planning for the future [23, 34, 35]. Financial concerns, including insurance, employment, and disability entitlements [23, 38], were also reported, with one strategy recording health care utilisation data and out-of-pocket costs to determine health economics cost-consequences [23]. These carer-identified needs changed across the illness continuum, reflecting the dynamic trajectory of caregiving for people with HGG.

While individual strategies may be of benefit in addressing one facet of need [47], the diversity of carer-identified needs outlined above highlights the importance of supportive interventions that can respond to emerging needs across the illness continuum [34, 38]. As such, supportive strategies should be based on regular, carer-driven needs assessment to identify in what domains the carer wants support. A recent study by Pointon and colleagues [53] reaffirmed this distinction between unmet needs and those that carers wanted support to address. Encouraging carers to identify when and where they require assistance not only provides an individualised response [35], but avoids unwanted and unnecessary interventions that could strain already limited health care resources [54]. These findings support the value-based approach in formulating and providing support to carers of people with HGG.

Translating beneficial trial strategies into clinical practice requires consideration of financial and resource allocation factors, and the capacity of health services to support implementation in the clinical setting. For example, a residential rehabilitation programme invested significant time and resources to benefit a small cohort of participants [46]. This finding is congruent with a systematic review of oncology carer interventions with only 11% of interventions less than 3 h in duration, potentially limiting their integration into clinical practice [55]. Conversely, a crude cost analysis of ‘I-CoPE’ estimated a mean cost of AUD$137 per dyad, with the strategy able to be incorporated into existing care-coordinator models of care [24]. Similarly, the ‘Family Strong’ programme was time-efficient and could be integrated into social work or care coordinator roles [35]. Future trial-based economic evaluations and more pragmatic preference-based approaches such as discrete choice experiments may be helpful in informing future practice and policy decisions to allocate resources.

## Limitations and ongoing or future research

There are several limitations in this review. The results of the published studies need to be interpreted with caution due to high risk of bias from missing data, measurement of outcomes, failure to adjust for confounders, or bias in the randomisation process. However, these limitations were expected, and therefore, this review focused on identifying carer-reported benefit to inform future research strategies. Grey literature was not included in this review and as such certain evaluations relevant to the topic of interest may not have been included. This review was also limited to studies published in English and conducted in Western countries which could limit generalisability of findings for carers in developing countries or from culturally diverse communities. Future research should also seek to include carers with a more diverse demographic, for example to explore the experiences of male partners, or children caring for parents with HGG.

There are several ongoing clinical trials which could provide further data to expand our knowledge of effective strategies for addressing supportive care needs. A ‘Care-IS’ RCT is currently in progress [56], as is a wait-list controlled trial combining eSNAP with a regular supportive phone review [57]. Similarly, further data are anticipated to expand on preliminary findings from recently published studies [41, 44]. Results from these RCTs and future research may contribute to identifying effective, efficient, and sustainable strategies to support carers of people with HGG.

## Conclusion

This systematic review evaluated supportive care strategies aimed at carers of people with HGG to determine the benefit to carers from their participation. Carers reported benefit from a range of strategies that provided educational and emotional support. Carer needs are diverse and change across the illness continuum, therefore supportive services should be tailored to individual needs and include both informational, practical, and emotional support. Supportive strategies also need to be feasible in relation to time and resource allocation, and economically sustainable to facilitate successful implementation into healthcare settings. Additional research is needed to adopt a value-based approach and strengthen the body of evidence on how to best support carers of people with HGG.

## References

[1] Ferlay J, Colombet M, Soerjomataram I, Mathers C, Parkin DM, Piñeros M (2019). Estimating the global cancer incidence and mortality in 2018: GLOBOCAN sources and methods. Int J Cancer.

[2] Louis DN, Perry A, Wesseling P, Brat DJ, Cree IA, Figarella-Branger D (2021). The 2021 WHO classification of tumors of the central nervous system: a summary. Neuro Oncol.

[3] Madsen K, Poulsen HS (2011). Needs for everyday life support for brain tumour patients’ relatives: systematic literature review. Eur J Cancer Care.

[4] Berger TR, Wen PY, Lang-Orsini M, Chukwueke UN (2022) World Health Organization 2021 classification of central nervous system tumors and implications for therapy for adult-type gliomas: a review. JAMA Oncol 10.1001/jamaoncol.2022.2844 36006639

[5] Piil K, Jakobsen J, Christensen KB, Juhler M, Guetterman TC, Fetters MD (2018). Needs and preferences among patients with high-grade glioma and their caregivers—a longitudinal mixed methods study. Eur J Cancer Care.

[6] McConigley R, Halkett G, Lobb E, Nowak A (2010). Caring for someone with high-grade glioma: a time of rapid change for caregivers. Palliat Med.

[7] Long A, Halkett GKB, Lobb EA, Shaw T, Hovey E, Nowak AK (2016). Carers of patients with high-grade glioma report high levels of distress, unmet needs, and psychological morbidity during patient chemoradiotherapy. Neuro-Oncol Practice.

[8] Halkett G, Lobb E, Shaw T, Sinclair M, Miller L, Hovey E (2017). Distress and psychological morbidity do not reduce over time in carers of patients with high-grade glioma. Support Care Cancer.

[9] Piil K, Nordentoft S, Larsen A, Jarden M (2019). Bereaved caregivers of patients with high-grade glioma: A systematic review. BMJ Support Palliat Care.

[10] Collins A, Lethborg C, Brand C, Gold M, Moore G, Sundararajan V (2014). The challenges and suffering of caring for people with primary malignant glioma: qualitative perspectives on improving current supportive and palliative care practices. BMJ Support Palliat Care.

[11] Halkett GKB, Lobb EA, Shaw T, Sinclair MM, Miller L, Hovey E (2018). Do carer’s levels of unmet needs change over time when caring for patients diagnosed with high-grade glioma and how are these needs correlated with distress?. Support Care Cancer.

[12] Francis SR, Hall EOC, Delmar C (2020). Ethical dilemmas experienced by spouses of a partner with brain tumour. Nurs Ethics.

[13] Coolbrandt A, Sterckx W, Clement P, Borgenon S, Decruyenaere M, De Vleeschouwer S (2015). Family Caregivers of patients with a high-grade glioma: a qualitative study of their lived experience and needs related to professional care. Cancer Nurs.

[14] Whisenant M (2011). Informal caregiving in patients with brain tumors. Oncol Nurs Forum.

[15] Boele FW, Grant R, Sherwood P (2017). Challenges and support for family caregivers of glioma patients. Br J Neurosci Nurs.

[16] Sherwood PR, Cwiklik M, Donovan HS (2016). Neuro-oncology family caregiving: review and directions for future research. CNS Oncol.

[17] Boele FW, Rooney AG, Bulbeck H, Sherwood P (2019) Interventions to help support caregivers of people with a brain or spinal cord tumour. Cochrane Database of Systematic Reviews 2019(7) 10.1002/14651858.CD012582.pub2 PMC6604115 31264707

[18] Boele FW, Hoeben W, Hilverda K, Lenting J, Calis AL, Sizoo EM (2013). Enhancing quality of life and mastery of informal caregivers of high-grade glioma patients: a randomized controlled trial. J Neurooncol.

[19] Locke DEC, Cerhan JH, Wu W, Malec JF, Clark MM, Rummans TA (2008). Cognitive rehabilitation and problem-solving to improve quality of life of patients with primary brain tumors: a pilot study. J Support Oncol.

[20] Reblin M, Ketcher D, Forsyth P, Mendivil E, Kane L, Pok J (2018). Outcomes of an electronic social network intervention with neuro-oncology patient family caregivers. J Neurooncol.

[21] Ownsworth T, Chan RJ, Jones S, Robertson J, Pinkham MB (2021). Use of telehealth platforms for delivering supportive care to adults with primary brain tumors and their family caregivers: a systematic review. Psychooncol.

[22] Piil K, Jakobsen J, Juhler M, Jarden M (2015). The feasibility of a brain tumour website. Eur J Oncol Nurs.

[23] Halkett GKB, Lobb EA, Miller L, Shaw T, Moorin R, Long A (2018). Feasibility testing and refinement of a supportive educational intervention for carers of patients with high-grade glioma - a pilot study. J Cancer Educ: the official journal of the American Association for Cancer Education.

[24] Philip J, Collins A, Staker J, Murphy M (2019). I-CoPE: a pilot study of structured supportive care delivery to people with newly diagnosied high-grade glioma and their carers. Neuro-Oncol Practice.

[25] Milbury K, Weathers S-P, Durrani S, Li Y, Whisenant M, Li J (2020). Online Couple-based meditation intervention for patients with primary or metastatic brain tumors and their partners: results of a pilot randomized controlled trial. J Pain Symptom Manage.

[26] Heinsch M, Cootes H, Wells H, Tickner C, Wilson J, Sultani G, et al (2021) Supporting friends and family of adults with a primary brain tumour: a systematic review. Health Soc Care Community 10.1111/hsc.13586 34633723

[27] Catt S, Chalmers A, Fallowfield L (2008). Psychosocial and supportive-care needs in high-grade glioma. Lancet Oncol.

[28] Page MJ, McKenzie JE, Bossuyt PM, Boutron I, Hoffmann TC, Mulrow CD (2021). The PRISMA 2020 statement: an updated guideline for reporting systematic reviews. Int J Surg.

[29] Girgis A, Lambert S, Lecathelinais C (2011). The supportive care needs survey for partners and caregivers of cancer survivors: development and psychometric evaluation. Psychooncol.

[30] Sterne JAC, Hernán MA, Reeves BC, Savović J, Berkman ND, Viswanathan M, Henry D, Altman DG, Ansari MT, Boutron I, Carpenter JR, Chan AW, Churchill R, Deeks JJ, Hróbjartsson A, Kirkham J, Jüni P, Loke YK, Pigott TD, Ramsay CR, Regidor D, Rothstein HR, Sandhu L, Santaguida PL, Schünemann HJ, Shea B, Shrier I, Tugwell P, Turner L, Valentine JC, Waddington H, Waters E, Wells GA, Whiting PF, Higgins JPT (2016) ROBINS-I: a tool for assessing risk of bias in non-randomized studies of interventions. BMJ 355 10.1136/bmj.i4919 PMC5062054 27733354

[31] Sterne JAC, Savović J, Page MJ, Elbers RG, Blencowe NS, Boutron I, Cates CJ, Cheng H-Y, Corbett MS, Eldridge SM, Hernán MA, Hopewell S, Hróbjartsson A, Junqueira DR, Jüni P, Kirkham JJ, Lasserson T, Li T, McAleenan A, Reeves BC, Shepperd S, Shrier I, Stewart LA, Tilling K, White IR, Whiting PF, Higgins JPT (2019) RoB 2: a revised tool for assessing risk of bias in randomised trials. BMJ 366 10.1136/bmj.l4898 31462531

[32] Hong QN, F‡bregues S, Bartlett G, Boardman FK, Cargo M, Dagenais P, et al (2018) The Mixed Methods Appraisal Tool (MMAT) version 2018 for information professionals and researchers. Educ Inf 34:285-91

[33] Popay J, Roberts H, Sowden A, Petticrew M, Arai L, Rodgers M, et al. Guidance on the conduct of narrative synthesis in systematic reviews: a product from the ESRC Methods Programme2006.

[34] Aoun SM, Deas K, Howting D, Lee G (2015) Exploring the support needs of family caregivers of patients with brain cancer using the CSNAT: a comparative study with other cancer groups. PLoS One 10(12) 10.1371/journal.pone.0145106 PMC4682982 26679505

[35] Dionne-Odom JN, Williams GR, Warren PP, Tims S, Huang C-HS, Taylor RA (2021). Implementing a clinic-based telehealth support service (FamilyStrong) for family caregivers of individuals with grade IV brain tumors. J Palliative Med.

[36] Milbury K, Mallaiah S, Mahajan A, Armstrong T, Weathers S-P, Moss KE (2018). Yoga program for high-grade glioma patients undergoing radiotherapy and their family caregivers. Integr Cancer Ther.

[37] Pace A, Villani V, Di Pasquale A, Benincasa D, Guariglia L, Ieraci S (2014). Home care for brain tumor patients. Neurooncol Pract.

[38] Page MS, Chang SM (2017). Creating a caregiver program in neuro-oncology. Neuro-Oncol Practice.

[39] Pompili A, Telera S, Villani V, Pace A (2014). Home palliative care and end of life issues in glioblastoma multiforme: results and comments from a homogeneous cohort of patients. Neurosurg Focus FOC.

[40] Wasilewski A, Serventi J, Ibegbu C, Wychowski T, Burke J, Mohile N (2020). Epilepsy education in gliomas: engaging patients and caregivers to improve care. Support Care Cancer.

[41] Boele FW, Weimer JM, Proudfoot J, Marsland AL, Armstrong TS, Given CW (2022). The effects of SmartCare(©) on neuro-oncology family caregivers' distress: a randomized controlled trial. Support Care Cancer.

[42] Reblin M, Ketcher D, Forsyth P, Mendivil E, Kane L, Pok J (2018). Feasibility of implementing an electronic social support and resource visualization tool for caregivers in a neuro-oncology clinic. Support Care Cancer.

[43] Cashman R, Bernstein LJ, Bilodeau D, Bovett G, Jackson B, Yousefi M (2007). Evaluation of an educational program for the caregivers of persons diagnosed with a malignant glioma. Canad Oncol Nurs J Revue Canad De Nurs Oncologique.

[44] Applebaum AJ, Roberts KE, Lynch K, Gebert R, Loschiavo M, Behrens M, et al (2022) A qualitative exploration of the feasibility and acceptability of Meaning-Centered Psychotherapy for Cancer Caregivers. Palliative and Supportive Care 1–7 10.1017/S1478951521002030 PMC9314455 35078552

[45] Catt S, Chalmers A, Critchley G, Fallowfield L (2012). Supportive follow-up in patients treated with radical intent for high-grade glioma. CNS Oncol.

[46] Nordentoft S, Dieperink KB, Johansson SD, Jarden M, Piil K (2021) Evaluation of a multimodal rehabilitative palliative care programme for patients with high‐grade glioma and their family caregivers. Scandinavian J Caring Sci 10.1111/scs.13019 34296773

[47] Milbury K, Li J, Weathers S-P, Mallaiah S, Armstrong T, Li Y (2019). Pilot randomized, controlled trial of a dyadic yoga program for glioma patients undergoing radiotherapy and their family caregivers. Neuro-Oncol Practice.

[48] Halkett GKB, Cormie P, McGough S, Zopf EM, Galvão DA, Newton RU (2021). Patients and carers' perspectives of participating in a pilot tailored exercise program during chemoradiotherapy for high grade glioma: a qualitative study. Eur J Cancer Care.

[49] Philip J, Collins A, Panozzo S, Staker J, Murphy M (2020). Mapping the nature of distress raised by patients with high-grade glioma and their family caregivers: a descriptive longitudinal study. Neuro-Oncol Practice.

[50] Aoun SM, Grande G, Howting D, Deas K, Toye C, Troeung L (2015). The impact of the carer support needs assessment tool (CSNAT) in community palliative care using a stepped wedge cluster trial. PloS one.

[51] Lobb E, Halkett G, Nowak A (2011). Patient and caregiver perceptions of communication of prognosis in high grade glioma. Psychooncol.

[52] Arber A, Hutson N, Guerrero D, Wilson S, Lucas C, Faithfull S (2010). Carers of patients with a primary malignant brain tumour: are their information needs being met?. Br J Neurosci Nurs.

[53] Pointon L, Grant R, Peoples S, Erridge S, Sherwood P, Klein M, Boele F (2021). P1206 Unmet needs and wish for support of informal caregivers of primary brain tumour patients. Neuro-Oncol.

[54] Applebaum AJ, Kent EE, Lichtenthal WG (2021). Documentation of caregivers as a standard of care. J Clin Oncol.

[55] Ferrell B, Wittenberg E (2017). A review of family caregiving intervention trials in oncology: family caregiving in oncology. CA: Cancer J Clin.

[56] Halkett GKB, Lobb EA, Miller L, Phillips JL, Shaw T, Moorin R (2015). Protocol for the Care-IS Trial: a randomised controlled trial of a supportive educational intervention for carers of patients with high-grade glioma (HGG). BMJ Open.

[57] Reblin M, Ketcher D, McCormick R, Barrios-Monroy V, Sutton SK, Zebrack B (2021). A randomized wait-list controlled trial of a social support intervention for caregivers of patients with primary malignant brain tumor. BMC Health Serv Res.

